# Sewage Saved

**Published:** 1854-07

**Authors:** 


SEWAGE SAVED.—A project, which gives every promise of success, is about to be tried in Glasgow, by which a very valuable portable solid manure will be extracted from the sewage of that city, which, it is estimated, may be sold, with a large profit to the company, at £2 a ton. Whenever this can be accomplished, the waste of our great cities will supersede the use of guano, and that now valuable commodity will at once cease to be worth the expense of carriage from Peru; while by having the sewage soil removed as rapidly as it accumulates, one of the main causes of city disease, will be at once removed.

---

IN woman, the disjunction of knowledge from thought, is especially strange and displeasing. I have known a woman of this kind from my earliest youth, and I have watched her through every period of her life. She is well acquainted with the dead, and with most living languages; is free from all vanity and affectation; never allows her studies to interfere with any domestic duty; yet her learning does not make her an interesting person. Though she has read the best, as well as the most difficult authors of all nations, she never writes a letter that gives one any extraordinary pleasure.—*W. Humboldt.*

